# Extracellular Vesicle Enriched miR-625-3p Is Associated with Survival of Malignant Mesothelioma Patients

**DOI:** 10.3390/jpm11101014

**Published:** 2021-10-09

**Authors:** Katja Goričar, Marija Holcar, Nina Mavec, Viljem Kovač, Metka Lenassi, Vita Dolžan

**Affiliations:** 1 Institute of Biochemistry and Molecular Genetics, Faculty of Medicine, University of Ljubljana, Vrazov trg 2, 1000 Ljubljana, Slovenia; katja.goricar@mf.uni-lj.si (K.G.); marija.holcar@mf.uni-lj.si (M.H.); nina.mavec@yahoo.com (N.M.); metka.lenassi@mf.uni-lj.si (M.L.); 2 Institute of Oncology Ljubljana, Zaloška 2, 1000 Ljubljana, Slovenia; vkovac@onko-i.si; 3 Faculty of Medicine, University of Ljubljana, Vrazov trg 2, 1000 Ljubljana, Slovenia

**Keywords:** mesothelioma, extracellular vesicles, miR-625, prognosis

## Abstract

Malignant mesothelioma (MM) is characterized by poor prognosis and short survival. Extracellular vesicles (EVs) are membrane-bound particles released from cells into various body fluids, and their molecular composition reflects the characteristics of the origin cell. Blood EVs or their miRNA cargo might serve as new minimally invasive biomarkers that would enable earlier detection of MM or treatment outcome prediction. Our aim was to evaluate miRNAs enriched in serum EVs as potential prognostic biomarkers in MM patients in a pilot longitudinal study. EVs were isolated from serum samples obtained before and after treatment using ultracentrifugation on 20% sucrose cushion. Serum EV-enriched miR-103-3p, miR-126-3p and miR-625-3p were quantified using qPCR. After treatment, expression of miR-625-3p and miR-126-3p significantly increased in MM patients with poor treatment outcome (*p* = 0.012 and *p* = 0.036, respectively). A relative increase in miR-625-3p expression after treatment for more than 3.2% was associated with shorter progression-free survival (7.5 vs. 19.4 months, HR = 3.92, 95% CI = 1.20–12.80, *p* = 0.024) and overall survival (12.5 vs. 49.1 months, HR = 5.45, 95% CI = 1.06–28.11, *p* = 0.043) of MM patients. Bioinformatic analysis showed enrichment of 33 miR-625-3p targets in eight biological pathways. Serum EV-enriched miR-625-3p could therefore serve as a prognostic biomarker in MM and could contribute to a more personalized treatment.

## 1. Introduction

Malignant mesothelioma (MM) is a rare aggressive malignancy of the pleura or the peritoneum that is mostly associated with exposure to asbestos [1]. Even though asbestos use has been banned in most countries, MM incidence is still rising due to a long latency period between asbestos exposure and development of MM [2]. As MM symptoms are often non-specific, diagnosis is usually made when the disease is already in the advanced stages [3]. MM is therefore characterized by poor prognosis and short survival [1,4].

MM treatment is often multimodal and includes chemotherapy, surgery, and radiation. Even though implementation of chemotherapy increased survival of MM patients, outcome is still limited [1,5,6]. Standard chemotherapy includes treatment with a combination of pemetrexed and cisplatin [7], and comparable results were shown for treatment with a combination of gemcitabine and cisplatin [1,8,9,10]. New treatment options based on immunotherapy or targeted treatment are currently extensively investigated in clinical trials (reviewed in [11,12,13]). Just recently, the combination of nivolumab and ipilimumab was approved by the U.S. Food and Drug Administration as a first-line treatment for unresectable pleural MM [14].

Several studies have tried to identify biomarkers that could improve the outcome of MM patients, mostly focusing on biomarkers for early diagnosis of MM. The best known MM biomarker is mesothelin; cell-surface glycoprotein increased in both tumor tissue and serum of MM patients [15,16,17,18]. Osteopontin and fibulin-3 were often proposed as additional MM biomarkers [18,19,20,21]. *MSLN* genetic variability also affects mesothelin levels and accounting for genetic factors can improve predictive ability of mesothelin [22,23,24,25]. On the other hand, fewer studies focused on identifying prognostic biomarkers in MM that would be able to predict treatment outcome. For example, increased mesothelin was associated with worse survival in a meta-analysis [26]. We have also identified several other pharmacogenetic biomarkers in drug transport, metabolism and target genes as well as DNA repair pathways that could help to predict response to chemotherapy based on clinical-pharmacogenetic models [27]. However, current biomarkers alone have limited sensitivity or specificity, preventing their widespread implementation in clinical practice [28]. The search for appropriate minimally invasive diagnostic and prognostic biomarkers in MM therefore continues, with studies focusing on composite biomarkers or new types of biomarkers.

MicroRNAs (miRNAs), endogenous, small, non-coding RNA sequences, which help to regulate gene expression at the post-transcriptional level, are emerging as important novel circulating biomarkers in cancer and other diseases [3]. In MM, several studies investigated miRNA expression in tumor tissue, blood cells, plasma or serum, pleural effusions or cell lines, and a number of miRNAs were implicated in MM pathogenesis and diagnosis (reviewed in [3,29,30]). Among them, miR-103-3p, miR-126-3p, and miR-625-3p were identified as suitable biomarkers in multiple studies [30,31,32,33,34,35,36,37,38,39,40,41,42]. In MM patients, miR-103-3p and miR-126 were downregulated compared to asbestos-exposed or healthy controls [30,31,32,33,34,35,36,37,38,39,40,41], while miR-625-3p was upregulated [30,42]. Some of the studies also suggested that a combination of a few miRNAs or their combination with mesothelin could serve as a better diagnostic biomarker [32,34,39,40]. On the other hand, the role of miRNAs in MM prognosis is not well established. So far, miR-126-3p expression was associated with shorter survival of MM patients in a few studies, alone or in combination with other miRNAs [35,41]. Additionally, increased circulating miR-625-3p expression after chemotherapy was associated with disease progression [43].

Recent studies have shown that miRNAs secreted by cells of primary tumors and metastatic sites into biofluids are often encapsulated within extracellular vesicles (EVs). EVs are phospholipid bilayer enclosed spherical nanoparticles, secreted by all cells investigated so far and reflecting their (patho)physiological state [44]. They can accumulate signals of disease or distress in form of nucleic acids, proteins, lipids and different metabolites and transport them to distant sites throughout the body. EVs are very heterogeneous in their biogenesis, release pathways, size, morphology, cargo and biophysical characteristics, and can be subdivided into exosomes, microvesicles and apoptotic bodies [45]. Their cargo is protected from degradation in the extracellular space and can be co-enriched from biofluids with EVs [46,47]. Changes in EV concentration or size as well as cargo composition were observed in different cancer types [48,49,50]. EVs or their cargo, e.g., miRNA, could therefore be used in liquid biopsy as diagnostic or prognostic cancer biomarkers, to assess disease progression, treatment response or resistance [51]. EV-miRNA cargo specifically has been shown to be actively involved in the regulation of diverse targets in recipient cells, among others regulating disease progression, metastasis and even sensitivity to specific drugs [52,53,54].

In MM, EVs secreted from cell lines were already shown to be enriched with proteins involved in different cellular pathways, including signalling, response to stress, angiogenesis, and metastasis [28,55,56]. Additionally, asbestos exposure modified EV cargo leading to gene expression changes in mesothelial cells [57]. However, only a few studies investigated EV-miRNA in MM to date [30,58,59]. The most abundant EV-bound miRNAs in MM were reported to be tumor suppressors [59]. EV-miR-103a-3p and miR-30e-3p were reported as candidate diagnostic markers in MM [58]. A meta-analysis of diagnostic value of miRNA in asbestos exposure and MM reported in the miRandola database found EVs-linked miR-126-3p and miR-103a-3p to be downregulated, while miR-625-3p, miR-29c-5p and miR-92a-3p were upregulated in MM [30].

MiRNAs miR-103-3p, miR-126-3p, and miR-625-3p were proposed as circulating diagnostic MM biomarkers in several studies and were previously also detected in EVs [58,60]. On the other hand, the prognostic role of EV-miRNA is largely unexplored. Therefore, the aim of the present pilot study was to evaluate serum EV-enriched miRNAs miR-103a-3p, miR-126-3p, and miR-625-3p as potential minimally invasive biomarkers of treatment outcome in patients with MM in a longitudinal setting.

## 2. Materials and Methods

### 2.1. Patients

We performed a pilot longitudinal study that included MM patients with pleural or peritoneal mesothelioma treated with chemotherapy at the Institute of Oncology Ljubljana in the period between 1 February 2009 and 31 July 2016. The diagnosis of pleural or peritoneal MM was established by thoracoscopy or laparoscopy, respectively. For all patients, MM diagnosis was confirmed histologically by an experienced pathologist. MM stage was determined according to the TNM staging system for pleural MM, while performance status was evaluated according to the Eastern Cooperative Oncology Group (ECOG) scores. Demographic and clinical data were obtained from medical records or assessed during a clinical interview. Written informed consent was obtained for all patients. The study was approved by the Slovenian Ethics Committee for Research in Medicine (41/02/09) and was carried out according to the Declaration of Helsinki.

Inclusion criteria were treatment in the specified period and availability of longitudinal samples. Among MM patients treated in this period, we selected 10 patients with poor treatment outcome and 10 patients with good treatment outcome based on overall survival (OS): patients with poor treatment outcome had OS of less than 15 months (10 patients), while patients with good treatment outcome had OS of more than 20 months (10 patients).

Serum samples of 20 MM patients were collected at two time points: at diagnosis and after completion of chemotherapy. Blood was sampled on the day of the last chemotherapy cycle, unless disease progression occurred before the last cycle. Serum samples were prepared within 4 h after blood sampling, aliquoted and stored at −20 °C. In total, 40 samples were evaluated.

### 2.2. Isolation of Small EVs with Sucrose Cushion Ultracentrifugation (sUC)

We used the established sUC method for enrichment of small EVs [61]. In short, sera aliquots were first defrosted on ice and centrifuged at 10,000× *g* for 20 min at 4 °C to remove any large extracellular particles. Next, 2 mL of 20% sucrose (sucrose (Merck Millipore, Burlington, MA, USA) in Dulbecco’s phosphate-buffered saline (dPBS, Sigma-Aldrich, St. Louis, MO, USA) was pipetted in polypropylene tubes (Beckman Coulter, Brea, CA, USA) and overlaid with diluted serum (1 mL of serum, mixed with 8.5 mL of particle-free dPBS). Samples were ultracentrifuged at 100,000× *g* for 135 min at 4 °C (MLA-55 rotor, Beckman Coulter, USA) and supernatant was aspirated from the tubes and walls of the tubes dried by low-lint highly absorbent paper. Finally, the pellet containing isolated EVs was fully resuspended in 200 µL of dPBS, mixed with 800 µL Tri-reagent (Sigma-Aldrich, USA), and stored at −20 °C.

### 2.3. Extraction of miRNA and Transcription to cDNA

Before miRNA extraction, 1 mL aliquots of frozen serum small EV-enriched samples, mixed with Tri-reagent, were defrosted on ice. 1 µL of MS2 RNA carrier (final concentration 0.8 µg/µL; Roche, Basel, Switzerland), 1 µL of spike-in (exogenous control, ath-miR-159a, final concentration 0.4 fM; Applied Biosystems, Waltham, MA, USA), and 200 µL of chloroform (Sigma-Aldrich, USA) were added to the samples and thoroughly mixed [61]. MiRNA was extracted using the miRNeasy Mini Kit (Qiagen, Hilden, Germany), according to the manufacturers’ instructions, with following adaptations of the protocol: (I) addition of extra 500 µL of RNase/DNase-free water and subsequent chloroform extraction after the first removal of aqueous phase from the chloroform-sample mixture, and (II) elution of miRNA from the column into DNA low binding tubes (Eppendorf, Hamburg, Germany) by two successive additions of 25 µL of RNase/DNase free water and centrifugations (15,000× *g*, 30 s). Samples of extracted miRNA were stored at −20 °C until batch reverse transcription of total isolated miRNA to cDNA for all samples. For this, TaqMan™ Advanced miRNA cDNA Synthesis Kit (Applied Biosystems, USA) was used, following the manufacturer’s instructions. cDNA samples were stored at −20 °C.

### 2.4. Quantitative Polymerase Chain Reaction (qPCR)

qPCR for miRNA expression analysis was performed using the TaqMan™ Advanced MicroRNA assays (Applied Biosystems, USA) on QuantStudio™ 7 Flex Real-Time PCR System (Applied Biosystems, USA). The analysis was performed using QuantStudio Software (Applied Biosystems, USA) and the miRNA levels were expressed as cycle threshold (Ct). Ct of spike-in (ath-miR-159a) was analyzed to evaluate the efficiency of miRNA isolation as well as transcription to cDNA, to exclude deviating samples according to the manufacturer’s instructions. All of the samples were also tested for hemolysis by analyzing miR-23a-3p and miR-451a expression. ∆Ct((miR-23a-3p)−(miR-451a)) ≥ 7 indicated hemolysis and led to exclusion of the sample from further analysis [62]. In addition to three miRNAs of interest (miR-103a-3p, miR-126-3p, miR-625-3p), two control miRNAs with reportedly stable expression in plasma or serum (let-7i-5p and miR-425-5p) [63,64] were analyzed. Expression of miRNAs of interest was normalized to the average expression of control miRNAs let-7i-5p and miR-425-5p. The relative expression of miRNAs was calculated as 2^−ΔCt^. Temporal changes in miRNA expression were assessed using relative change, defined as the difference of miRNA expression after treatment and at diagnosis, divided by its value at diagnosis.

### 2.5. Bioinformatic Analysis of miR-625-3p Targets

Experimentally validated miR-625-3p targets were obtained using miRTarBase (2020 update) [65]. Interaction network predicting the relationship between miR-625-3p target genes and genes correlating with target genes was obtained using GeneMania based on automatically selected weighting method [66]. We used gProfiler for functional enrichment analysis based on Gene Ontology (GO), Kyoto Encyclopedia of Genes and Genomes (KEGG), Reactome, and WikiPathways as well as Transfac, miRTarBase, Human Protein Atlas, CORUM, and Human Phenotype Ontology databases [67]. To account for multiple comparisons, multiple testing correction based on g:SCS algorithm was used.

### 2.6. Statistical Analysis

Continuous variables were described using median and interquartile range (25–75%), and categorical variables were described using frequencies. For continuous dependent variables, the nonparametric Mann–Whitney test was used to compare the distribution among different groups, while Fisher’s exact test was used to compare the distribution of categorical variables. For related samples, the nonparametric Wilcoxon signed-rank test was used for comparison of continuous variables in different time points. For the differentiation between MM patients with poor and good treatment outcome, a receiver operating characteristic (ROC) curve analysis was used to determine the specificity, sensitivity and area under the curve (AUC). Cutoff values were selected as values with the highest sum of specificity and sensitivity.

In survival analysis, progression-free survival (PFS) was defined as the time from diagnosis to the day of documented disease progression or death from any cause, and OS was defined as the time from diagnosis to death from any cause. Patients without progression or death at the time of the analysis were censored at the date of the last follow-up. Kaplan–Meier analysis was used to calculate median survival or follow-up time. Univariable and multivariable Cox regression was used to calculate the hazard ratios (HR) and the 95% confidence intervals (CIs). Clinical variables used for adjustment in multivariable survival analysis were selected using stepwise forward conditional selection.

All statistical analyses were carried out by IBM SPSS Statistics version 21.0 (IBM Corporation, Armonk, NY, USA). All statistical tests were two-sided and the level of significance was set at 0.05.

## 3. Results

### 3.1. Patient Characteristics

The final study group consisted of 18 MM patients. Two patients were excluded from the analysis because their samples obtained at diagnosis did not pass the quality control for spike-in and/or hemolysis levels. Patients’ clinical characteristics are presented in Table 1. Among them, 8 (44.4%) patients had poor treatment outcome, while 10 (55.6%) patients had good treatment outcome. In total, 17 (94.4%) patients had pleural and 1 (5.6%) patient had peritoneal MM. The median follow-up time was 30.8 months. The relative change of miRNA expression during treatment was only evaluated in 17 patients (8 (47.1%) patients with poor and 9 (52.9%) with good outcome), as one sample obtained at the end of chemotherapy was excluded due to hemolysis.

Among all MM patients, 12 (66.7%) were treated with gemcitabine and cisplatin doublet chemotherapy, while 6 (33.3%) received pemetrexed and cisplatin doublet chemotherapy. There were no significant differences in treatment outcome between both chemotherapy regimens (*p* = 0.638).

### 3.2. Comparison of Serum EV-Enriched miRNA Expression at Diagnosis and after Treatment

First, we evaluated if the expression of target serum EV-enriched miRNAs changes after treatment with chemotherapy in MM patients (Table 2, Figure S1). The expression of EV-enriched miR-126-3p increased after treatment in 12 (70.6%) patients (*p* = 0.035, Table 2, Figure S1c). Expression of EV-enriched miR-625-3p and miR-103a-3p did not differ significantly after treatment (Figure S1a,b, respectively).

When patients were stratified according to outcome, the expression of EV-enriched miR-625-3p and miR-126-3p was significantly increased after treatment in patients with poor outcome (*p* = 0.012 and *p* = 0.036, respectively, Table 2). Expression of EV-enriched miR-625-3p increased after treatment in all 8 patients with poor outcome, while EV-enriched miR-126-3p expression increased in 6 (75.0%) patients with poor outcome (Figure S1d,f, respectively). On the other hand, no differences between miRNA expression at diagnosis and after treatment were observed in patients with good outcome (Table 2).

### 3.3. Differentiation between MM Patients with Poor and Good Treatment Outcome Based on Serum EV-Enriched miRNA Expression

There were no significant differences in the expression of serum EV-enriched miRNAs collected at diagnosis between MM patients with poor and good treatment outcome (Table 3). On the other hand, a relative change in EV-enriched miR-625-3p expression over time could discriminate between patients with poor and good treatment outcome. After treatment, miR-625-3p expression increased in patients with poor outcome (median 85.2%) and decreased in patients with good outcome (median −17.5%), and the difference was statistically significant (*p* = 0.036). AUC for miR-625-3p was 0.806 (0.588–1.000) (*p* = 0.034). At the cutoff value of 3.2% with the highest sum of specificity and sensitivity, sensitivity was 0.667 and specificity was 1.000. Relative change of EV-enriched miR-103a-3p or miR-126-3p expression after treatment was not associated with treatment outcome (Table 3). The relative change of EV-enriched miRNA expression did not differ between different chemotherapy regimens (*p* = 0.884 for miR-625-3p, *p* = 0.733 for miR-103a-3p, and *p* = 0.525 for miR-126-3p).

### 3.4. Survival Analysis

For MM patients with poor outcome, median PFS was 6.9 (5.8–7.5) months and median OS was 10.0 (7.7–12.5) months. For MM patients with good outcome, median PFS was 19.4 (14.9–23.2) months and median OS was 29.4 (27.3–49.1) months. Among clinical characteristics, higher C-reactive protein (CRP) level was an important predictor of shorter OS (HR = 1.01, 95% CI = 1.00–1.04, *p* = 0.029) and was therefore used for adjustment in multivariable analyses. The chemotherapy regimen was not a significant predictor of OS (HR = 0.21, 95% CI = 0.03–1.67, *p* = 0.141).

Serum EV-enriched miRNA expression at diagnosis was not associated with survival of MM patients (Table S1). On the other hand, a higher relative change in miR-625-3p was associated with both worse PFS (HR = 1.02, 95% CI = 1.00–1.04, *p* = 0.044) and worse OS (HR = 1.02, 95% CI = 1.00–1.05, *p* = 0.045). The association remained significant after adjustment for clinical variables in multivariable analysis (PFS: HR = 1.02, 95% CI = 1.00–1.04, *p* = 0.046; OS: HR = 1.03, 95% CI = 1.00–1.05, *p* = 0.042; Table S1). EV-enriched miR-103a-3p or miR-126-3p was not associated with survival of MM patients.

**Figure 1:**
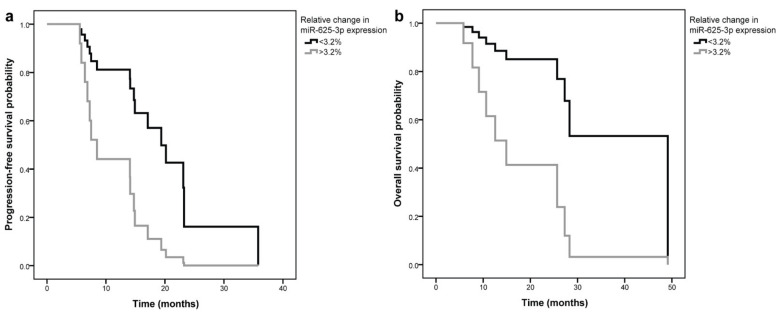
Relative change in serum EV-enriched miR-625-3p expression and progression free survival (**a**) and overall survival (**b**) of malignant mesothelioma patients. EV: extracellular vesicles.

Patients were then stratified according to cutoff values obtained from comparison between patients with poor and good outcome (Table 3), and the association with PFS and OS was assessed (Table 4). A relative increase in EV-enriched miR-625-3p expression after treatment for more than 3.2% was associated with significantly shorter PFS (7.5 compared to 19.4 months, Figure 1a). The difference was significant both in univariable analysis (HR = 3.92, 95% CI = 1.20–12.80, *p* = 0.024) and after adjustment for CRP levels at diagnosis (HR = 4.13, 95% CI = 1.25–13.65, *p* = 0.020). Similarly, a relative increase in miR-625-3p expression after treatment for more than 3.2% was associated with significantly shorter OS (12.5 vs. 49.1 months, Figure 1b). The difference was again significant both in univariable analysis (HR = 5.45, 95% CI = 1.06–28.11, *p* = 0.043) and after adjustment for CRP levels at diagnosis (HR = 6.32, 95% CI = 1.18–33.99, *p* = 0.032).

### 3.5. Bioinformatic Analysis of miR-625-3p Targets

miRTarBase listed 33 experimentally confirmed miR-625-3p targets. Only one of them, mitogen-activated protein kinase kinase 6 (MAP2K6), was experimentally confirmed using reporter assay, Western blot and qPCR, while other targets were only confirmed using next-generation sequencing.

**Figure 2:**
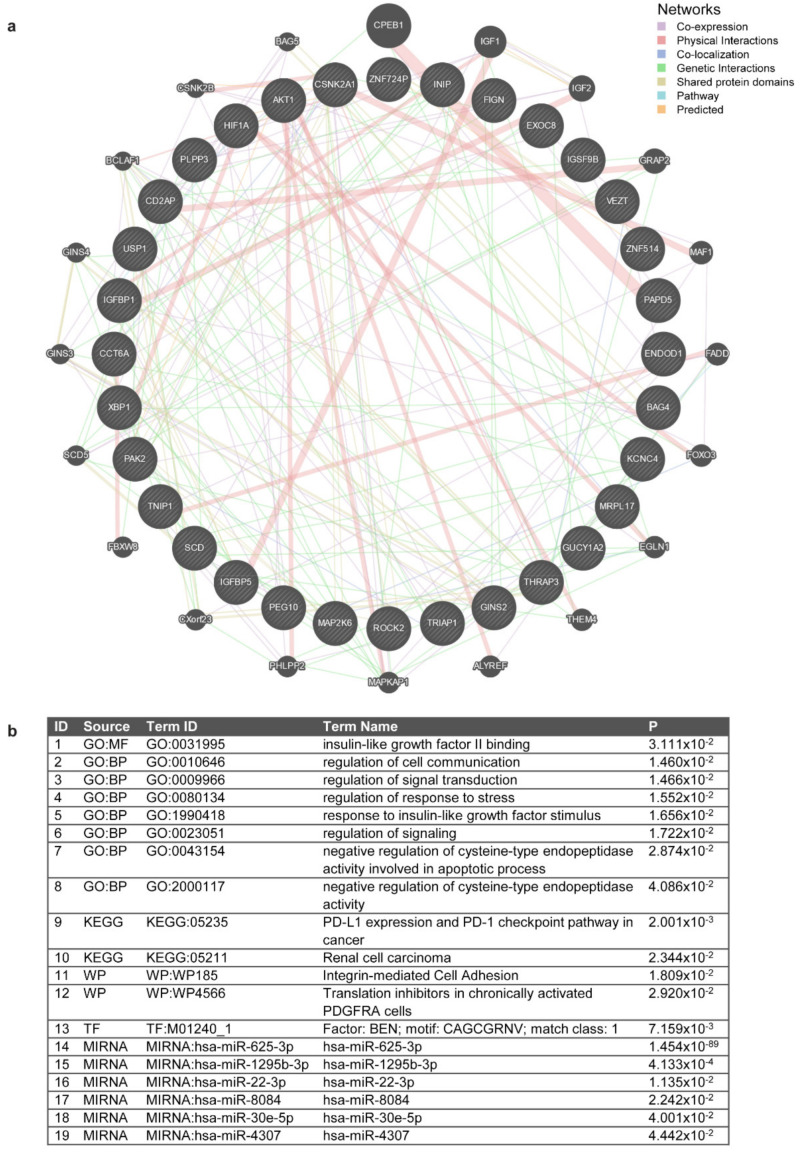
(**a**) Experimentally confirmed miR-625-3p targets and their interactions based on co-expression, physical interactions, co-localization, genetic interactions, shared protein domains, and predicted interactions. MiR-625-3p target genes are presented in the inner circle, while the outer circle shows other associated genes based on GeneMania analysis. Target gene *c7orf65* was not included in GeneMania. (**b**) gProfiler pathway enrichment analysis: biological processes and pathways linked to miR-625-3p target genes, term names, codes and significance level. BP: biological process; GO: gene ontology; KEGG: Kyoto Encyclopedia of Genes and Genomes; MF: molecular function; TF: transcription factor; WP: WikiPathways.

Using GeneMania, we evaluated co-expression, physical interactions, co-localization, genetic interactions, shared protein domains, and predicted interactions of miR-625-3p target genes. Interaction network revealed several associations between miR-625-3p target genes, as well as 20 additional interacting genes (Figure 2a).

A gProfiler analysis showed that the identified miR-625-3p target genes were significantly associated not only with miR-625-3p but also with five other miRNAs, especially miR-1295b-3p (Figure 2b). After GO and KEGG enrichment analysis of miR-625-3p target genes, eight GO (seven biological pathways and one molecular function) and two KEGG terms were enriched in this gene set. The most significant pathway after enrichment was PD-L1 expression and PD-1 checkpoint pathway in cancer (KEGG:05235). The most significant GO biological process terms were regulation of cell communication (GO:0010646) and regulation of signal transduction (GO:0009966). The only significant GO molecular function term was insulin-like growth factor II binding (GO:0031995). Additionally, two WikiPathways were also enriched among miR-625-3p target genes. Based on Transfac data, BEN transcription factor binding motif was significantly enriched in our data set. A detailed description of all significant pathways and processes and their significance level is represented in Figure 2b.

## 4. Discussion

In the present pilot longitudinal study, we investigated expression of candidate miRNAs enriched in serum small EVs as potential prognostic biomarkers in MM. After treatment with platinum-based chemotherapy, expression of serum EV-enriched miR-625-3p and miR-126-3p significantly increased only in MM patients with poor treatment outcome. A relative increase in EV-enriched miR-625-3p expression after treatment was associated with significantly shorter survival and could be used as a prognostic biomarker in MM.

The most important result of our study is the association of serum EV-enriched miR-625-3p with treatment outcome and survival of MM patients. Expression of serum EV-enriched miR-625-3p significantly increased after treatment with platinum-based chemotherapy in MM patients with poor treatment outcome, while a nonsignificant decrease was observed in MM patients with good outcome. Relative change in serum EV-enriched miR-625-3p expression over time could discriminate between patients with poor and good treatment outcome with high specificity. If serum EV-enriched miR-625-3p expression after treatment increased for more than 3.2%, MM patients had significantly shorter PFS and OS, even after adjustment for clinical parameters. In previous studies, circulating plasma miR-625-3p was generally upregulated in MM compared to healthy controls, but the results are conflicting [30,34,42]. In the only study evaluating EVs in MM, no differences in miR-625-3p expression were observed compared to controls, while its prognostic potential was not assessed [58]. In a longitudinal study that investigated plasma miR-625-3p in MM before and after cisplatin-based chemotherapy, expression increased in patients with progressive disease [43], which is consistent with our results. The combination of increased miR-625-3p and decreased long noncoding RNA (lncRNA) GAS5 expression could distinguish between patients with good or poor outcome, even though only GAS5 was associated with overall survival [43]. In concordance with our results, increased tumor miR-625-3p expression was associated with worse response to oxaliplatin and oxaliplatin resistance in colorectal cancer [68,69], emphasizing the association between miR-625-3p and response to platinum compounds. Additionally, increased tumor miR-625-3p expression was also significantly associated with tumor relapse in esophageal small cell carcinoma [70]. High tumor miR-625-3p expression was observed in thyroid and clear cell renal cell carcinoma [71,72]. Increased miR-625-3p expression was associated with poor prognosis, tumor proliferation, migration or invasion in various cancers [71,72,73].

Despite strong evidence that miR-625-3p may be associated with unfavorable prognosis, miR-625 was also observed to be downregulated in serum, plasma or tissue in some cancer types; however, most of these studies did not specify whether they investigated the expression of miR-625-3p or miR-625-5p [74,75,76,77]. Decreased miR-625 expression was associated with shorter survival of esophageal squamous cell carcinoma [74], and its expression increased in non-small cell lung cancer after surgery and in acute lymphoblastic leukemia in remission [75,77]. Some studies also suggested miR-625 might suppress cell proliferation, migration and invasion, and identified a number of different miR-625 target genes [78,79,80,81]. Based on data from miRTarBase, these target genes were associated with miR-625-5p, suggesting these studies were investigating miR-625-5p and that there are important differences in the biological roles of these two miRNAs as they might regulate different pathways or be differentially regulated themselves. For example, different lncRNAs were identified as potential regulators of miR-625-3p or miR-625-5p expression [43,81,82]. Additionally, several isoforms of miR-625-3p with potentially differential expression were reported, but their role is not yet established [43].

We therefore tried to further elucidate the role of miR-625-3p using bioinformatic analysis. According to the miRTarBase database, 33 miR-625-3p targets were experimentally confirmed, and the interaction network revealed several interactions between them as well as some common interacting genes. However, *MAP2K6* was the only target confirmed with strong evidence. Mitogen-activated protein kinase kinase MAP2K6 is involved in p38 phosphorylation in response to stress and thus affects apoptosis and cell cycle [69]. Intrachromosomal rearrangements of this gene were previously observed in MM [83]. MAP2K6 was identified as a direct mediator of miR-625-3p associated oxaliplatin resistance in colorectal cancer [69], and it was proposed that miR-625-3p and *MAP2K6* could even be used to guide treatment selection [84]. Evaluation of *MAP2K6* expression would therefore also be of great interest in MM.

A number of other miR-625-3p target genes identified by bioinformatic analysis were previously implicated in MM or in response to asbestos, further confirming this miRNA might play an important role. For example, asbestos exposure was associated with modified expression of THRAP3 and PEG10 [85], XBP1 [86], TNIP1 and PLPP3 [87]. AKT1 and its signalling pathway were implicated in various processes in MM, including resistance to cisplatin [88]. ROCK2 was overexpressed in MM tumor tissue and implicated in the Hippo signalling pathway [89]. Additionally, HIF1A and hypoxia were also associated with MM, for example, with proliferation and inflammation as well as histological type [90].

Pathway enrichment analysis showed miR-625-3p target genes are involved in several different processes. In GO analysis, seven biological process terms were enriched, most significantly regulation of cell communication and regulation of signal transduction, while insulin-like growth factor II binding was the only significant molecular function term. Insulin-like growth factor II mRNA-binding protein 3 was already proposed as a biomarker for distinguishing between MM and benign mesothelial proliferations [91]. Identified miR-625-3p target genes were associated with five additional miRNAs, especially miR-1295b-3p; however, not much is known about this miRNA.

Interestingly, among two significant KEGG pathways, the most significant was PD-L1 expression and PD-1 checkpoint pathway in cancer. Immune checkpoint proteins programmed cell death protein (PD-1) and programmed cell death 1 ligand 1 (PD-L1) were extensively investigated in MM in the past few years due to their potential as targets in immunotherapy [11,12,13]. However, there is great interindividual variability in response to anti-PD-1 and anti-PD-L1 treatment, and the success of treatment with a single immune checkpoint inhibitor is limited [12]. Tumor PD-L1 expression is not a sufficient biomarker for identification of MM patients that could benefit from immunotherapy, and novel biomarkers are needed [12,13]. Importantly, PD-L1 expression was also identified in EVs [28], and EVs could therefore be a potential additional biomarker guiding immunotherapy personalization. Further studies investigating PD-L1 in EVs, also in combination with EV-enriched miR-625-3p, are therefore needed.

In our study, we also evaluated the potential biomarker role of EV-enriched miR-126-3p and miR-103-3p. Expression of EV-enriched miR-126-3p significantly increased only in MM patients with poor treatment outcome. However, the relative expression change after treatment was not associated with outcome or survival. Multiple studies identified miR-126-3p as a standalone or composite diagnostic biomarker that can discriminate between MM patients and controls, both in serum or plasma and in tissue samples [30,33,34,35,36,37,38,39,40,41], suggesting this miRNA has an important role in MM pathogenesis. Studies show that miR-126 plays a role in the regulation of mitochondrial metabolism, and is associated with oxidative stress, hypoxia and autophagy pathways [92,93]. However, miR-126-3p was not a suitable screening biomarker for early detection of MM in prediagnostic plasma samples [94]. Serum or tissue miR-126-3p expression was previously associated with shorter survival of MM patients [35,41], but this association was not confirmed in all studies [36,37]. Interestingly, miR-126 might be involved in cell communication, as exosomal transfer of miR-126 was associated with anti-tumor response and angiogenesis in MM cell lines [60]. On the other hand, EV-miR-126-3p expression did not differ among MM patients and controls [58]. Further studies focusing on change of miR-126-3p expression after treatment are therefore needed to better evaluate miR-126-3p as a prognostic biomarker in MM.

Serum EV-enriched miR-103-3p was not a good prognostic biomarker in MM in our study. So far, studies have shown miR-103-3p is downregulated, especially in cellular fraction of peripheral blood samples in MM patients compared to asbestos-exposed controls and was proposed as a diagnostic biomarker, standalone or in combination with mesothelin [30,31,32,33]. However, it did not enable early detection of MM in prediagnostic plasma samples [94]. In plasma EVs, miR-103-3p was downregulated compared to asbestos-exposed controls and the combination of miR-103a-3p and miR-30e-3p was the best diagnostic marker [58]. Patients with higher expression of EV-miR-103-3p tended to have longer overall survival, but the difference was not statistically significant [58]. However, expression change after treatment was not evaluated. Other studies did not investigate miR-103-3p as a potential prognostic biomarker in MM.

The vast majority of previous studies investigated circulating miRNAs, while we focused on miRNAs enriched in EVs. Previous studies suggested that miRNA in cancer-derived EVs might be a more suitable biomarker than circulating miRNAs, as EVs protect miRNAs from degradation. Additionally, EVs may be enriched with miRNAs reflecting their origin cell that are therefore more specific [95]. Furthermore, since miRNAs are often present in serum at very low concentrations, their EV-related enrichment in the sample can also improve sensitivity. On the other hand, enrichment of EVs from serum samples introduces an additional step in the miRNA-extraction protocol, which could present a drawback in larger studies or in clinical practice. Standardized methods for EV and miRNA extraction and the use of appropriate exogenous and endogenous controls for quality control and normalization are also needed to enable direct comparison between studies. Many different approaches for normalization of miRNA expression were previously proposed; however, there is still no universally accepted method of normalization for EVs-miRNAs, which can contribute to differences between studies [41,96,97,98]. It is also important to point out that our study did not focus exclusively on vesicle-enclosed miRNA, as miRNAs can also bind to the surface of EVs and we did not treat samples of isolated EVs with RNAse A prior to RNA extraction.

The main limitation of our study is the small sample size however, it was designed as a proof-of-concept study. Due to the strict inclusion criteria and quality-control exclusion criteria, we were nevertheless able to identify the most differentially expressed serum EV-enriched miRNAs. Furthermore, one of the key advantages of our study was its longitudinal design that enabled measurement of temporal changes in EV-miRNA expression after treatment. Still, studies including more MM patients are needed to validate our results and evaluate the usefulness of EV-enriched miR-625-3p in practical use in treatment prognosis in MM. Another limitation of our study is that no data on *BAP1* mutation status or other germline mutations were available. Inherited loss-of-function mutations in DNA repair genes or other tumor suppressor genes, especially *BAP1*, were associated with increased MM risk, but also improved survival, particularly following platinum-based chemotherapy [99,100,101]. In the future, evaluation of the combined effect of EV-enriched miRNA and germline mutations on survival could enable identification of better prognostic biomarkers. Additionally, even though previous studies showed that miRNAs miR-126-3p and miR-625-3p expression is deregulated in MM tumor tissue [33,35,36,37,38,40,42], studies evaluating EVs derived from MM tumor tissue are lacking. Further larger studies on EVs in MM, focusing also on biomarker combinations, are therefore needed to confirm our results.

## 5. Conclusions

Biological fluids are an ideal source for liquid biopsies, a complementary tool to traditional tissue biopsies that may aid in early disease discovery, monitoring of disease progression or success of the treatment [102,103]. Peripheral circulating venous blood is an easily accessible body fluid and still the most widely used source for biomarkers of a variety of the diseases, including different cancers [102,104,105]. However, differences in results and study design currently limit the translation of miRNA biomarkers to clinical practice. Our results and the results of other studies suggest EVs should also be considered as potential diagnostic or prognostic biomarkers in MM, especially in patients receiving platinum-based chemotherapy. Monitoring EV-enriched miR-625-3p expression might contribute to the prediction of treatment outcome and selection of therapy in MM patients, especially for subsequent lines of systemic treatment. For MM patients with predicted poor treatment outcome with platinum-based chemotherapy, novel systemic treatment approaches might be implemented sooner, while additional surgical treatment might be used for MM patients with predicted good treatment outcome. Additionally, EVs could in the future also be used in novel treatment approaches, for example, modulating miRNA expression [59,106].

In conclusion, serum EV-enriched miR-625-3p was associated with treatment outcome and survival of MM patients in our proof-of-concept study and might serve as a prognostic biomarker. EVs or their cargo might therefore contribute to a more personalized treatment that could improve the prognosis of MM patients.

## Figures and Tables

**Table 1:** Clinical characteristics of malignant mesothelioma patients (*n* = 18).

Characteristic	Category/Unit	*n* (%)
Gender	Male	10 (55.6)
	Female	8 (44.4)
Age	Years, Median (25–75%)	68.5 (59.8–72.5)
Stage	I	4 (22.2)
	II	2 (11.1)
	III	8 (44.4)
	IV	3 (16.7)
	Peritoneal	1 (5.6)
Histological type	Epithelioid	12 (66.7)
	Biphasic	3 (16.7)
	Sarcomatoid	3 (16.7)
ECOG performance status	0	4 (22.2)
	1	8 (44.4)
	2	6 (33.3)
Asbestos exposure	Not exposed	5 (27.8)
	Exposed	13 (72.2)
Smoking	Non-smokers	11 (61.1)
	Smokers	7 (38.9)
CRP	mg/L, Median (25–75%)	15.5 (2.8–46.5)
Chemotherapy	Gemcitabine + cisplatin	12 (66.7)
	Pemetrexed + cisplatin	6 (33.3)
PFS	Months, Median (25–75%)	14.1 (7.2–20.2)
OS	Months, Median (25–75%)	27.3 (12.5–29.4)
Follow-up time	Months, Median (25–75%)	30.8 (23.4–30.8)

CRP: C-reactive protein; ECOG: Eastern Cooperative Oncology Group; EV: extracellular vesicles; OS: overall survival; PFS: progression-free survival.

**Table 2:** Comparison of expression of serum EV-enriched miRNAs at diagnosis and after treatment in malignant mesothelioma patients.

	miRNA	At Diagnosis Relative Expression Median (25–75%)	After Treatment Relative Expression Median (25–75%)	*p*
All patients (*n* = 17)	miR-625-3p	0.05 (0.01–0.13)	0.07 (0.03–0.15)	0.227
	miR-103a-3p	0.40 (0.34–0.47)	0.39 (0.32–0.48)	0.981
	miR-126-3p	45.73 (38.30–74.96)	68.05 (46.17–101.77)	0.035
Poor outcome (*n* = 8)	miR-625-3p	0.06 (0.02–0.13)	0.11 (0.08–0.21)	0.012
	miR-103a-3p	0.39 (0.28–0.42)	0.37 (0.28–0.48)	0.889
	miR-126-3p	55.01 (038.03–72.06)	78.81 (55.58–140.34)	0.036
Good outcome (*n* = 9)	miR-625-3p	0.04 (0.01–0.14)	0.04 (0.01–0.05)	0.173
	miR-103a-3p	0.43 (0.34–0.50)	0.40 (0.33–0.51)	0.953
	miR-126-3p	44.46 (38.76–77.33)	51.51 (37.69–94.09)	0.374

EV: extracellular vesicles.

**Table 3:** Expression of serum EV-enriched miRNAs and treatment outcome of malignant mesothelioma patients and ROC curve analysis.

	miRNA	Poor Outcome Median (25–75%)	Good Outcome Median (25–75%)	*p*	AUC (95% CI)	*p*	Cutoff	Sensitivity	Specificity
At diagnosis	miR-625-3p	0.06 (0.02–0.13)	0.05 (0.01–0.13)	0.897	0.556 (0.273–0.838)	0.700	0.01	0.333	0.875
(*n* = 18)	miR-103a-3p	0.39 (0.28–0.42)	0.45 (0.36–0.54)	0.146	0.681 (0.414–0.947)	0.211	0.47	0.444	1.000
	miR-126-3p	55.01 (38.03–72.06)	45.09 (39.81–76.49)	0.965	0.514 (0.221–0.807)	0.923	46.28	0.667	0.925
Change (%)	miR-625-3p	85.2 (25.8–565.9)	−17.5 (−82.8–150.6)	0.036	0.806 (0.588–1.000)	0.034	3.2	0.667	1.000
(*n* = 17)	miR-103a-3p	1.6 (-13.9–25.2)	−10.5 (−29.8–37.1)	0.888	0.528 (0.242–0.814)	0.847	−16.7	0.333	0.875
	miR-126-3p	16.1 (1.7–175.97)	20.7 (−24.8–86.8)	0.606	0.583 (0.297–0.869)	0.564	−8.7	0.333	1.000

AUC: area under the curve; CI: confidence interval; EV: extracellular vesicles; ROC: receiver operating characteristic.

**Table 4:** Relative change in expression of serum EV-enriched miRNAs and progression-free survival (PFS) and overall survival (OS) of malignant mesothelioma patients.

miRNA	PFS						OS					
	<Cutoff Months, Median (25–75%)	>Cutoff Months, Median (25–75%)	HR (95% CI)	*p*	HR (95% CI)_adj_	P_adj_	<Cutoff Months, Median (25–75%)	>Cutoff Months, Median (25–75%)	HR (95% CI)	*p*	HR (95% CI)_adj_	P_adj_
miR-625-3p	19.4 (14.9–23.2)	7.5 (6.4–14.7)	3.92 (1.2–12.8)	0.024	4.13 (1.25–13.65)	0.020	49.1 (27.3–49.1)	12.5 (9.1–28.3)	5.45 (1.06–28.11)	0.043	6.32 (1.18–33.99)	0.032
miR-103a-3p	14.9 (5.8–17.1)	14.1 (7.2–19.4)	1.84 (0.52–6.56)	0.348	1.76 (0.47–6.56)	0.403	27.3 (5.8–49.1)	25.7 (10.6–28.3)	1.50 (0.30–7.37)	0.621	1.35 (0.26–6.95)	0.716
miR-126-3p	19.4 (14.9–23.2)	8.5 (6.9–17.1)	1.89 (0.53–6.76)	0.327	2.90 (0.71–11.89)	0.140	27.3 (27.3–27.3)	25.7 (10.6–49.1)	2.40 (0.29–19.60)	0.416	7.77 (0.72–84.17)	0.092

adj: adjusted for C-reactive protein levels at diagnosis. CI: confidence interval; EV: extracellular vesicles; HR: hazard ratio.

## Data Availability

All the data are presented within the article and in Supplementary Materials. Any additional information is available on request from the corresponding author.

## Supplementary Materials

The following are available online at www.mdpi.com/article/10.3390/jpm11101014/s1, Figure S1: Comparison of expression of serum EV-enriched miRNAs at diagnosis and after treatment in each malignant mesothelioma patient in the whole study group (a: miR-625-3p, b: miR-103a-3p, c: miR-126-3p; dark blue: increased expression, light blue: decreased expression) and in each patient with poor treatment outcome (d: miR-625-3p, e: miR-103a-3p, f: miR-126-3p; dark green: increased expression, light green: decreased expression), Table S1: Expression of serum EV-enriched miRNAs and progression-free survival (PFS) and overall survival (OS) of malignant mesothelioma patients.
