# Supplementary material for: Extracellular Vesicle Enriched miR-625-3p Is Associated with Survival of Malignant Mesothelioma Patients
Source: J Pers Med. 2021 Oct 9;11(10):1014. doi: 10.3390/jpm11101014 (PMC8538530; doi:10.3390/jpm11101014)
Supplement: Supplementary file 1 [file jpm-11-01014-s001.zip › jpm-1398117-supplementary.pdf]

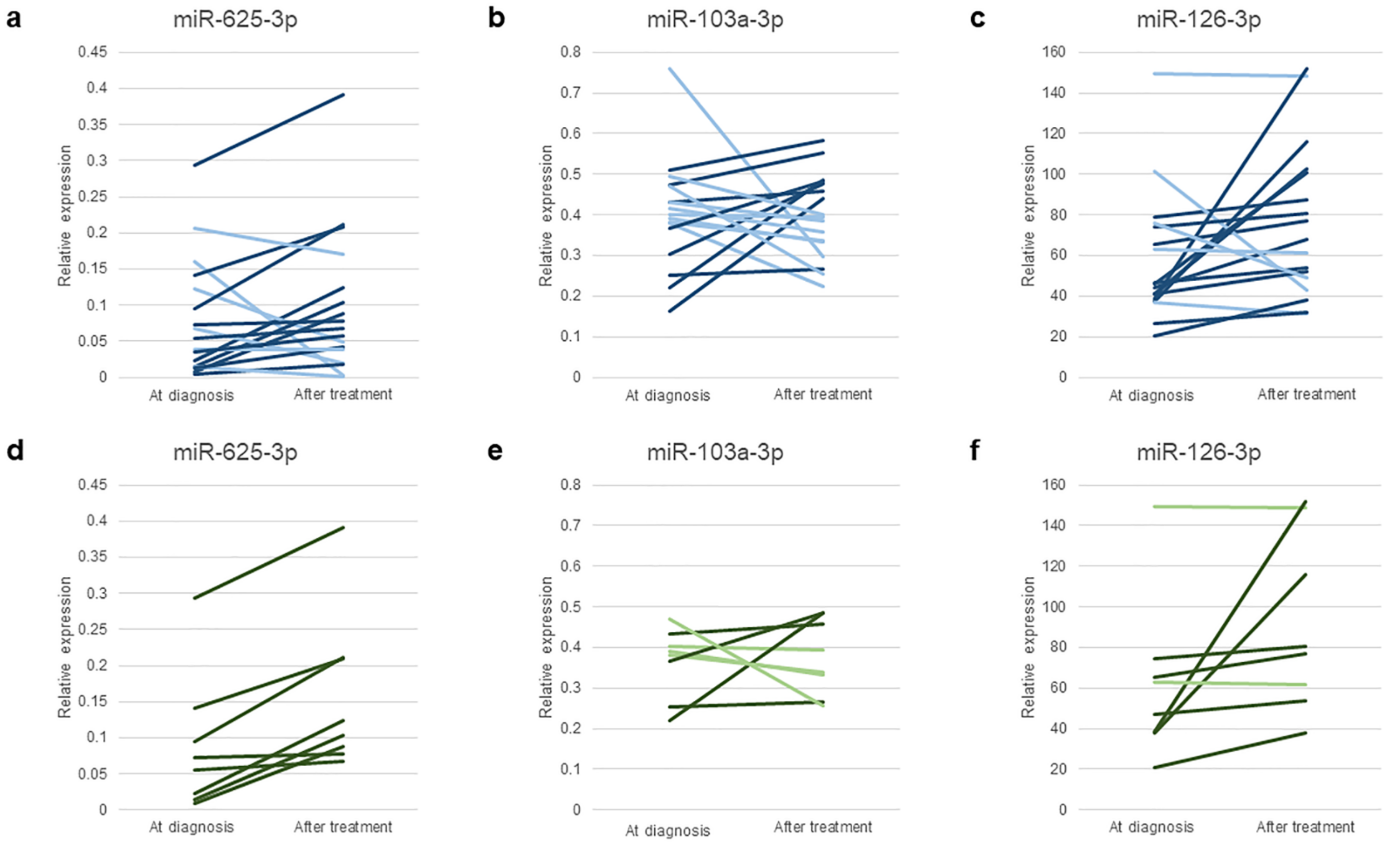

Figure S1: Comparison of expression of serum EV-enriched miRNAs at diagnosis and after treatment in each malignant mesothelioma patient in the whole study group (a: miR-625-3p, b: miR-103a-3p, c: miR-126-3p; dark blue: increased expression, light blue: decreased expression) and in each patient with poor treatment outcome (d: miR-625-3p, e: miR-103a-3p, f: miR-126-3p; dark green: increased expression, light green: decreased expression).
EV: extracellular vesicles

Table S1: Expression of serum EV-enriched miRNAs and progression-free survival (PFS) and overall survival (OS) of malignant mesothelioma patients.

	**miRNA**	**PFS**				**OS**			
		**HR (95% CI)**	**P**	**HR (95% CI)$_{adj}$**	**P$_{adj}$**	**HR (95% CI)**	**P**	**HR (95% CI)$_{adj}$**	**P$_{adj}$**
At diagnosis (N=18)	miR-625-3p	0.33 (0.00-340.58)	0.755	1.74 (0.00-4333.43)	0.890	0.06 (0.00-393.16)	0.535	3.60 (0.00-69206.36)	0.799
	miR-103a-3p	0.35 (0.01-8.61)	0.520	0.26 (0.01-7.00)	0.421	0.28 (0.01-14.32)	0.529	0.13 (0.00-7.02)	0.319
	miR-126-3p	1.01 (0.99-1.02)	0.533	1.01 (0.99-1.02)	0.531	1.01 (0.99-1.03)	0.192	1.01 (0.99-1.03)	0.233
Change (%)* (N=17)	miR-625-3p	1.02 (1.00-1.04)	**0.044**	1.02 (1.00-1.04)	**0.046**	1.02 (1.00-1.05)	**0.045**	1.03 (1.00-1.05)	**0.042**
	miR-103a-3p	0.99 (0.91-1.07)	0.714	0.99 (0.91-1.07)	0.742	1.01 (0.92-1.10)	0.847	1.02 (0.93-1.11)	0.725
	miR-126-3p	1.01 (0.96-1.07)	0.642	1.02 (0.96-1.09)	0.482	1.00 (0.93-1.08)	0.994	1.03 (0.95-1.12)	0.458

adj: adjusted for C-reactive protein levels at diagnosis
*HR values are reported for a difference of 10%
CI: confidence interval; EV: extracellular vesicles; HR: hazard ratio
